# Supplementary material for: The Roles of MTRR and MTHFR Gene Polymorphisms in Colorectal Cancer Survival
Source: Nutrients. 2022 Nov 1;14(21):4594. doi: 10.3390/nu14214594 (PMC9658674; doi:10.3390/nu14214594)
Supplement: Supplementary file 1 [file nutrients-14-04594-s001.zip › nutrients-1970065-supplementary.pdf]

Table S1. Association Between *MTRR* SNPs and Colorectal Cancer Overall, Disease-Free and CRC Specific Survival Assuming an Additive Mode of Inheritance.

	Position [a]	Minor/Major allele (MAF)	All			Colon			Rectum		
			HR (95%CI)	*P*	P_{ACT} [d]	HR (95%CI)	*P*	P_{ACT} [d]	HR (95%CI)	*P*	P_{ACT} [d]
**Overall survival [b]**											
rs1801394	7870973	A/G (0.430)	0.77 (0.60,0.98)	0.036	0.491	0.66 (0.47,0.91)	0.011	0.206	1.00 (0.65,1.53)	0.980	1.000
rs13181011	7874424	C/T (0.196)	1.27 (0.92,1.76)	0.154	0.875	1.54 (1.04,2.28)	0.031	0.430	0.88 (0.46,1.68)	0.703	1.000
rs7730643	7875963	G/A (0.168)	1.08 (0.76,1.53)	0.683	1.000	1.03 (0.63,1.67)	0.912	1.000	1.13 (0.66,1.91)	0.661	1.000
rs326121	7876288	C/T (0.239)	0.99 (0.74,1.32)	0.925	1.000	0.93 (0.63,1.38)	0.712	1.000	0.94 (0.57,1.56)	0.808	1.000
rs3776467	7876315	C/T (0.247)	0.70 (0.50,0.98)	0.036	0.483	0.69 (0.45,1.06)	0.091	0.744	0.65 (0.37,1.14)	0.134	0.899
rs326123	7876861	G/A (0.402)	1.16 (0.90,1.50)	0.261	0.968	1.21 (0.88,1.66)	0.236	0.935	1.12 (0.70,1.81)	0.635	1.000
rs326124	7877178	A/G (0.178)	1.11 (0.78,1.59)	0.563	1.000	1.29 (0.81,2.08)	0.285	0.950	0.85 (0.48,1.53)	0.588	1.000
rs1532268	7878179	A/G (0.349)	1.03 (0.78,1.36)	0.828	1.000	1.21 (0.85,1.71)	0.289	0.951	0.90 (0.56,1.44)	0.650	1.000
rs7703033	7879950	A/G (0.334)	1.09 (0.84,1.42)	0.518	1.000	1.18 (0.84,1.67)	0.346	0.968	0.88 (0.56,1.38)	0.589	1.000
rs6555501	7879983	C/T (0.429)	1.03 (0.81,1.32)	0.794	1.000	0.99 (0.73,1.34)	0.927	1.000	1.01 (0.65,1.58)	0.953	1.000
rs162031	7880287	T/C (0.210)	1.05 (0.76,1.44)	0.767	1.000	0.98 (0.65,1.48)	0.915	1.000	1.21 (0.71,2.05)	0.491	1.000
rs162033	7880835	T/C (0.419)	0.99 (0.78,1.26)	0.932	1.000	0.89 (0.65,1.21)	0.439	0.982	1.07 (0.69,1.67)	0.762	1.000
rs161871	7884389	G/A (0.166)	0.93 (0.66,1.30)	0.659	1.000	0.85 (0.56,1.30)	0.456	0.982	1.08 (0.57,2.05)	0.819	1.000
rs162040	7887478	C/A (0.153)	0.92 (0.62,1.35)	0.652	1.000	0.77 (0.46,1.30)	0.331	0.968	1.17 (0.62,2.24)	0.629	1.000
rs3776455	7896511	G/A (0.344)	0.89 (0.68,1.17)	0.410	0.993	0.75 (0.53,1.08)	0.122	0.810	1.13 (0.74,1.72)	0.587	1.000
rs10380	7897191	T/C (0.095)	0.90 (0.60,1.34)	0.596	1.000	0.70 (0.42,1.18)	0.181	0.887	1.56 (0.73,3.36)	0.252	0.985
rs9332	7900712	T/C (0.113)	0.82 (0.55,1.22)	0.323	0.975	0.64 (0.39,1.08)	0.093	0.733	1.33 (0.62,2.86)	0.465	1.000
**Disease-free survival [b]**											
rs1801394	7870973	A/G (0.430)	0.72 (0.57,0.90)	0.005	0.105	0.60 (0.44,0.81)	0.001	0.024	0.98 (0.67,1.44)	0.909	1.000
rs13181011	7874424	C/T (0.196)	1.35 (1.00,1.82)	0.048	0.556	1.62 (1.11,2.35)	0.012	0.213	0.93 (0.54,1.62)	0.809	1.000
rs7730643	7875963	G/A (0.168)	1.10 (0.80,1.50)	0.565	1.000	0.99 (0.64,1.53)	0.952	1.000	1.15 (0.72,1.86)	0.554	1.000
rs326121	7876288	C/T (0.239)	0.94 (0.72,1.22)	0.634	1.000	0.86 (0.60,1.23)	0.406	0.988	1.04 (0.67,1.60)	0.874	1.000
rs3776467	7876315	C/T (0.247)	0.72 (0.53,0.96)	0.026	0.388	0.71 (0.49,1.03)	0.074	0.697	0.67 (0.41,1.10)	0.116	0.863
rs326123	7876861	G/A (0.402)	1.05 (0.83,1.33)	0.683	1.000	1.06 (0.79,1.42)	0.694	1.000	1.02 (0.68,1.55)	0.918	1.000
rs326124	7877178	A/G (0.178)	1.41 (1.01,1.97)	0.043	0.529	1.63 (1.04,2.56)	0.032	0.434	1.14 (0.66,1.97)	0.632	1.000
rs1532268	7878179	A/G (0.349)	1.15 (0.90,1.46)	0.275	0.981	1.33 (0.97,1.83)	0.079	0.701	0.89 (0.59,1.34)	0.574	1.000
rs7703033	7879950	A/G (0.334)	1.13 (0.89,1.44)	0.311	0.981	1.29 (0.94,1.77)	0.120	0.818	0.92 (0.62,1.37)	0.697	1.000
rs6555501	7879983	C/T (0.429)	1.03 (0.83,1.29)	0.783	1.000	1.05 (0.80,1.40)	0.718	1.000	1.04 (0.70,1.53)	0.855	1.000
rs162031	7880287	T/C (0.210)	0.86 (0.64,1.15)	0.297	0.985	0.76 (0.52,1.13)	0.175	0.900	1.03 (0.63,1.67)	0.905	1.000
rs162033	7880835	T/C (0.419)	0.99 (0.79,1.23)	0.898	1.000	0.95 (0.72,1.26)	0.718	1.000	1.14 (0.77,1.70)	0.520	1.000
rs161871	7884389	G/A (0.166)	0.93 (0.68,1.26)	0.620	1.000	0.79 (0.54,1.16)	0.224	0.941	1.30 (0.76,2.24)	0.331	0.996
rs162040	7887478	C/A (0.153)	0.71 (0.49,1.02)	0.062	0.632	0.58 (0.35,0.96)	0.034	0.449	0.85 (0.48,1.53)	0.593	1.000
rs3776455	7896511	G/A (0.344)	0.78 (0.61,0.99)	0.044	0.532	0.62 (0.44,0.86)	0.005	0.102	1.07 (0.72,1.57)	0.750	1.000
rs10380	7897191	T/C (0.095)	0.82 (0.56,1.20)	0.304	0.983	0.71 (0.45,1.14)	0.156	0.881	1.51 (0.75,3.02)	0.248	0.983
rs9332	7900712	T/C (0.113)	0.77 (0.54,1.12)	0.173	0.921	0.64 (0.40,1.01)	0.057	0.611	1.46 (0.75,2.83)	0.268	0.987
**CRC specific survival [c]**											
rs1801394	7870973	A/G (0.438)	0.73 (0.49,1.07)	0.106	0.769	0.42 (0.23,0.78)	0.006	0.108	1.17 (0.65,2.10)	0.597	1.000
rs13181011	7874424	C/T (0.197)	1.40 (0.87,2.23)	0.164	0.878	1.84 (1.03,3.29)	0.039	0.396	0.68 (0.25,1.80)	0.433	0.998
rs7730643	7875963	G/A (0.173)	1.03 (0.62,1.71)	0.901	1.000	0.62 (0.26,1.46)	0.274	0.931	1.46 (0.73,2.94)	0.287	0.987
rs326121	7876288	C/T (0.240)	0.99 (0.66,1.49)	0.958	1.000	0.82 (0.43,1.56)	0.537	1.000	0.97 (0.51,1.86)	0.934	1.000
rs3776467	7876315	C/T (0.257)	0.73 (0.45,1.16)	0.181	0.895	0.57 (0.28,1.18)	0.131	0.777	0.62 (0.27,1.43)	0.259	0.984
rs326123	7876861	G/A (0.398)	1.06 (0.71,1.58)	0.789	1.000	1.06 (0.63,1.79)	0.836	1.000	0.95 (0.45,2.03)	0.896	1.000
rs326124	7877178	A/G (0.175)	1.38 (0.77,2.46)	0.278	0.952	2.54 (0.96,6.74)	0.061	0.525	0.80 (0.35,1.83)	0.596	1.000
rs1532268	7878179	A/G (0.353)	1.03 (0.69,1.53)	0.882	1.000	1.04 (0.61,1.78)	0.878	1.000	0.91 (0.47,1.79)	0.792	1.000
rs7703033	7879950	A/G (0.326)	1.04 (0.70,1.54)	0.842	1.000	1.49 (0.82,2.73)	0.192	0.875	0.67 (0.32,1.38)	0.275	0.985
rs6555501	7879983	C/T (0.427)	1.16 (0.81,1.68)	0.423	0.991	1.37 (0.83,2.24)	0.216	0.902	1.06 (0.50,2.23)	0.883	1.000
rs162031	7880287	T/C (0.203)	0.71 (0.40,1.23)	0.221	0.927	0.35 (0.13,0.92)	0.033	0.355	1.16 (0.55,2.46)	0.697	1.000
rs162033	7880835	T/C (0.418)	1.16 (0.78,1.71)	0.463	0.995	1.28 (0.76,2.13)	0.352	0.960	1.14 (0.50,2.60)	0.750	1.000
rs161871	7884389	G/A (0.174)	1.16 (0.74,1.84)	0.518	1.000	1.21 (0.67,2.18)	0.526	1.000	1.35 (0.60,3.02)	0.471	0.998
rs162040	7887478	C/A (0.154)	0.84 (0.46,1.54)	0.571	1.000	0.45 (0.17,1.22)	0.115	0.746	1.36 (0.56,3.27)	0.498	0.999
rs3776455	7896511	G/A (0.350)	0.94 (0.63,1.40)	0.759	1.000	0.77 (0.43,1.37)	0.372	0.964	1.29 (0.72,2.29)	0.391	0.997
rs10380	7897191	T/C (0.101)	1.27 (0.76,2.12)	0.354	0.982	1.10 (0.56,2.15)	0.786	1.000	2.49 (1.03,6.03)	0.043	0.544
rs9332	7900712	T/C (0.122)	1.24 (0.75,2.06)	0.405	0.990	1.15 (0.60,2.20)	0.684	1.000	2.04 (0.83,5.04)	0.123	0.866

Abbreviations: CRC, colorectal cancer; *MTRR*, methionine synthase reductase; SNPs, single nucleotide polymorphisms; HR, hazard ratio; MAF, minor allele frequency. [a] SNPs locations were mapped according to the NCBI build 36 coordinates. [b] Hazard ratio calculated in reference to the allele underlined. Cox proportional hazard model adjusted for sex, age at diagnosis, stage at diagnosis, race, marital status and MSI status. [c] Hazard ratio calculated in reference to the allele underlined. Cox

proportional hazard model adjusted for sex, age at diagnosis, stage at diagnosis, drink status, marital status and MSI status. [d] P-values were adjusted for multiple comparisons using a modification of P_{ACT} for correlated tests developed by Conneely and Boehnke [35].

Table S2. Association Between *MTHFR* SNPs and Colorectal Cancer Overall, Disease-Free and CRC-Specific Survival Assuming an Additive Mode of Inheritance.

	Position [a]	Minor/Major allele (MAF)	All			Colon			Rectum		
			HR (95%CI)	P	P_{ACT} [d]	HR (95%CI)	P	P_{ACT} [d]	HR (95%CI)	P	P_{ACT} [d]
**Overall survival [b]**											
rs4846048	11846252	G/A (0.295)	1.15 (0.89,1.49)	0.297	0.972	1.26 (0.91,1.76)	0.167	0.874	0.82 (0.51,1.32)	0.416	0.999
rs2184226	11847436	G/A (0.069)	0.77 (0.46,1.30)	0.323	0.978	0.62 (0.29,1.33)	0.218	0.924	0.91 (0.43,1.95)	0.814	1.000
rs3737966	11847759	G/A (0.391)	1.24 (0.97,1.57)	0.082	0.723	1.30 (0.95,1.77)	0.102	0.761	1.06 (0.70,1.61)	0.789	1.000
rs4846049	11850365	T/G (0.327)	1.37 (1.06,1.76)	0.017	0.289	1.48 (1.08,2.04)	0.016	0.271	1.07 (0.67,1.70)	0.773	1.000
rs1476413	11852300	A/G (0.266)	1.34 (1.02,1.76)	0.038	0.484	1.38 (0.96,1.98)	0.080	0.733	1.27 (0.79,2.05)	0.323	0.996
rs1801131	11854476	C/A (0.311)	1.37 (1.06,1.78)	0.015	0.264	1.50 (1.08,2.07)	0.014	0.244	1.06 (0.67,1.68)	0.810	1.000
rs12121543	11854671	A/C (0.243)	1.33 (1.01,1.75)	0.045	0.537	1.53 (1.07,2.19)	0.021	0.330	0.95 (0.57,1.57)	0.835	1.000
rs1801133	11856378	T/C (0.318)	0.90 (0.70,1.16)	0.416	0.989	0.88 (0.63,1.22)	0.430	0.987	1.09 (0.68,1.74)	0.735	1.000
rs1572151	11857711	G/A (0.068)	0.80 (0.47,1.36)	0.414	0.993	0.65 (0.30,1.41)	0.280	0.960	0.92 (0.43,1.99)	0.838	1.000
rs4846052	11857951	T/C (0.397)	1.28 (1.00,1.64)	0.050	0.554	1.36 (0.99,1.87)	0.061	0.642	1.07 (0.70,1.64)	0.769	1.000
rs2066471	11860458	A/G (0.174)	1.22 (0.89,1.68)	0.221	0.947	1.36 (0.89,2.07)	0.156	0.863	0.96 (0.56,1.65)	0.889	1.000
rs13306567	11860465	C/G (0.062)	1.26 (0.83,1.92)	0.285	0.975	1.61 (0.94,2.78)	0.086	0.745	0.71 (0.30,1.66)	0.427	1.000
rs7533315	11860683	T/C (0.240)	1.08 (0.80,1.45)	0.617	1.000	1.15 (0.77,1.71)	0.496	0.964	0.89 (0.55,1.44)	0.627	1.000
rs9651118	11862214	C/T (0.254)	0.80 (0.59,1.08)	0.144	0.871	0.77 (0.53,1.11)	0.154	0.874	0.88 (0.50,1.54)	0.650	1.000
rs7553194	11864149	A/G (0.095)	1.38 (0.93,2.06)	0.114	0.822	1.19 (0.72,1.97)	0.493	0.981	2.52 (1.19,5.34)	0.016	0.276
rs13306561	11865804	C/T (0.158)	1.39 (1.01,1.92)	0.045	0.528	1.43 (0.95,2.17)	0.086	0.752	1.35 (0.75,2.40)	0.316	0.995
**Disease-free survival [b]**											
rs4846048	11846252	G/A (0.295)	1.05 (0.83,1.34)	0.675	1.000	1.17 (0.86,1.59)	0.314	0.973	0.84 (0.55,1.29)	0.432	0.999
rs2184226	11847436	G/A (0.069)	0.80 (0.49,1.30)	0.362	0.987	0.77 (0.39,1.50)	0.438	0.985	0.87 (0.41,1.83)	0.715	1.000
rs3737966	11847759	G/A (0.391)	1.20 (0.96,1.49)	0.114	0.824	1.26 (0.95,1.68)	0.106	0.786	1.11 (0.76,1.62)	0.583	1.000
rs4846049	11850365	T/G (0.327)	1.19 (0.94,1.50)	0.155	0.898	1.29 (0.96,1.73)	0.097	0.759	1.01 (0.67,1.53)	0.962	1.000
rs1476413	11852300	A/G (0.266)	1.15 (0.89,1.49)	0.280	0.981	1.20 (0.87,1.67)	0.270	0.957	1.03 (0.67,1.59)	0.901	1.000
rs1801131	11854476	C/A (0.311)	1.19 (0.94,1.50)	0.160	0.905	1.28 (0.94,1.72)	0.113	0.802	1.03 (0.69,1.54)	0.878	1.000
rs12121543	11854671	A/C (0.243)	1.13 (0.87,1.46)	0.359	0.989	1.28 (0.92,1.78)	0.143	0.863	0.83 (0.53,1.30)	0.405	0.999
rs1801133	11856378	T/C (0.318)	0.90 (0.71,1.15)	0.392	0.988	0.91 (0.67,1.24)	0.551	1.000	0.89 (0.58,1.36)	0.595	1.000
rs1572151	11857711	G/A (0.068)	0.77 (0.47,1.26)	0.304	0.980	0.70 (0.36,1.37)	0.299	0.969	0.93 (0.44,1.97)	0.853	1.000
rs4846052	11857951	T/C (0.397)	1.11 (0.89,1.40)	0.356	0.989	1.19 (0.89,1.60)	0.249	0.945	1.01 (0.69,1.47)	0.954	1.000
rs2066471	11860458	A/G (0.174)	1.05 (0.78,1.41)	0.739	1.000	1.17 (0.80,1.71)	0.426	0.985	0.84 (0.51,1.38)	0.498	1.000
rs13306567	11860465	C/G (0.062)	1.14 (0.77,1.68)	0.529	1.000	1.31 (0.77,2.24)	0.316	0.972	1.03 (0.53,2.02)	0.935	1.000
rs7533315	11860683	T/C (0.240)	0.95 (0.72,1.25)	0.713	1.000	1.03 (0.72,1.49)	0.869	1.000	0.82 (0.53,1.27)	0.364	0.998
rs9651118	11862214	C/T (0.254)	0.98 (0.75,1.27)	0.858	1.000	0.89 (0.64,1.25)	0.505	1.000	1.13 (0.71,1.81)	0.599	1.000
rs7553194	11864149	A/G (0.095)	1.23 (0.86,1.78)	0.258	0.979	1.11 (0.71,1.73)	0.660	1.000	1.79 (0.90,3.55)	0.098	0.823
rs13306561	11865804	C/T (0.158)	1.25 (0.93,1.68)	0.134	0.865	1.26 (0.87,1.83)	0.228	0.931	1.35 (0.82,2.21)	0.238	0.981
**CRC specific survival [c]**											
rs4846048	11846252	G/A (0.294)	1.80 (1.22,2.65)	0.003	0.057	2.07 (1.21,3.54)	0.008	0.128	1.09 (0.55,2.16)	0.811	1.000
rs2184226	11847436	G/A (0.069)	1.07 (0.51,2.23)	0.867	1.000	0.66 (0.15,2.89)	0.583	1.000	0.84 (0.29,2.50)	0.760	1.000
rs3737966	11847759	G/A (0.386)	2.17 (1.49,3.14)	<0.0001	0.003	3.13 (1.82,5.39)	<0.0001	0.003	1.33 (0.71,2.49)	0.378	0.996
rs4846049	11850365	T/G (0.325)	2.09 (1.43,3.07)	0.0002	0.005	3.20 (1.84,5.60)	<0.0001	0.002	1.39 (0.71,2.72)	0.343	0.993
rs1476413	11852300	A/G (0.267)	2.66 (1.76,4.03)	<0.0001	0.003	3.17 (1.76,5.71)	0.0001	0.003	2.66 (1.34,5.27)	0.005	0.105
rs1801131	11854476	C/A (0.306)	2.06 (1.40,3.04)	0.0003	0.007	3.30 (1.89,5.76)	<0.0001	0.003	1.24 (0.62,2.47)	0.538	1.000
rs12121543	11854671	A/C (0.241)	2.27 (1.47,3.49)	0.0002	0.005	3.36 (1.88,6.01)	<0.0001	0.003	1.38 (0.63,3.02)	0.423	0.998
rs1801133	11856378	T/C (0.319)	0.52 (0.33,0.82)	0.005	0.086	0.28 (0.13,0.60)	0.001	0.021	0.85 (0.43,1.67)	0.630	1.000
rs1572151	11857711	G/A (0.069)	1.17 (0.59,2.35)	0.652	1.000	0.71 (0.18,2.80)	0.624	1.000	0.93 (0.33,2.64)	0.892	1.000
rs4846052	11857951	T/C (0.394)	1.95 (1.37,2.78)	0.0002	0.005	2.76 (1.63,4.69)	0.0002	0.005	1.24 (0.69,2.25)	0.470	0.999
rs2066471	11860458	A/G (0.177)	2.43 (1.50,3.94)	0.0003	0.007	2.41 (1.21,4.80)	0.012	0.181	2.66 (1.18,6.02)	0.019	0.314
rs13306567	11860465	C/G (0.058)	0.94 (0.49,1.79)	0.845	1.000	1.66 (0.71,3.88)	0.241	0.924	0.22 (0.04,1.09)	0.064	0.672
rs7533315	11860683	T/C (0.243)	2.03 (1.32,3.13)	0.001	0.021	2.09 (1.07,4.09)	0.032	0.363	1.55 (0.82,2.94)	0.180	0.940
rs9651118	11862214	C/T (0.255)	0.75 (0.47,1.20)	0.230	0.933	0.78 (0.41,1.48)	0.449	0.983	1.19 (0.46,3.10)	0.724	1.000
rs7553194	11864149	A/G (0.094)	2.00 (1.13,3.52)	0.017	0.237	2.25 (1.12,4.53)	0.023	0.299	2.22 (0.77,6.42)	0.141	0.895
rs13306561	11865804	C/T (0.153)	1.43 (0.91,2.25)	0.119	0.800	2.38 (1.26,4.50)	0.007	0.120	0.72 (0.31,1.70)	0.452	0.999

Abbreviations: CRC, colorectal cancer; HR, hazard ratio; *MTHFR*, methylenetetrahydrofolate reductase; MAF, minor allele frequency. [a] SNPs locations were mapped according to the NCBI build 36 coordinates. [b] Cox proportional hazard model adjusted for sex, age at diagnosis, stage at diagnosis, race, marital status and MSI status. [c] Cox proportional hazard model adjusted for sex, age at diagnosis, stage at diagnosis, drink status, folate taken status, marital status and MSI status. [d] P-values were adjusted for multiple comparisons using a modification of P_{ACT} for correlated tests developed by Conneely and Boehnke [35].

A

B

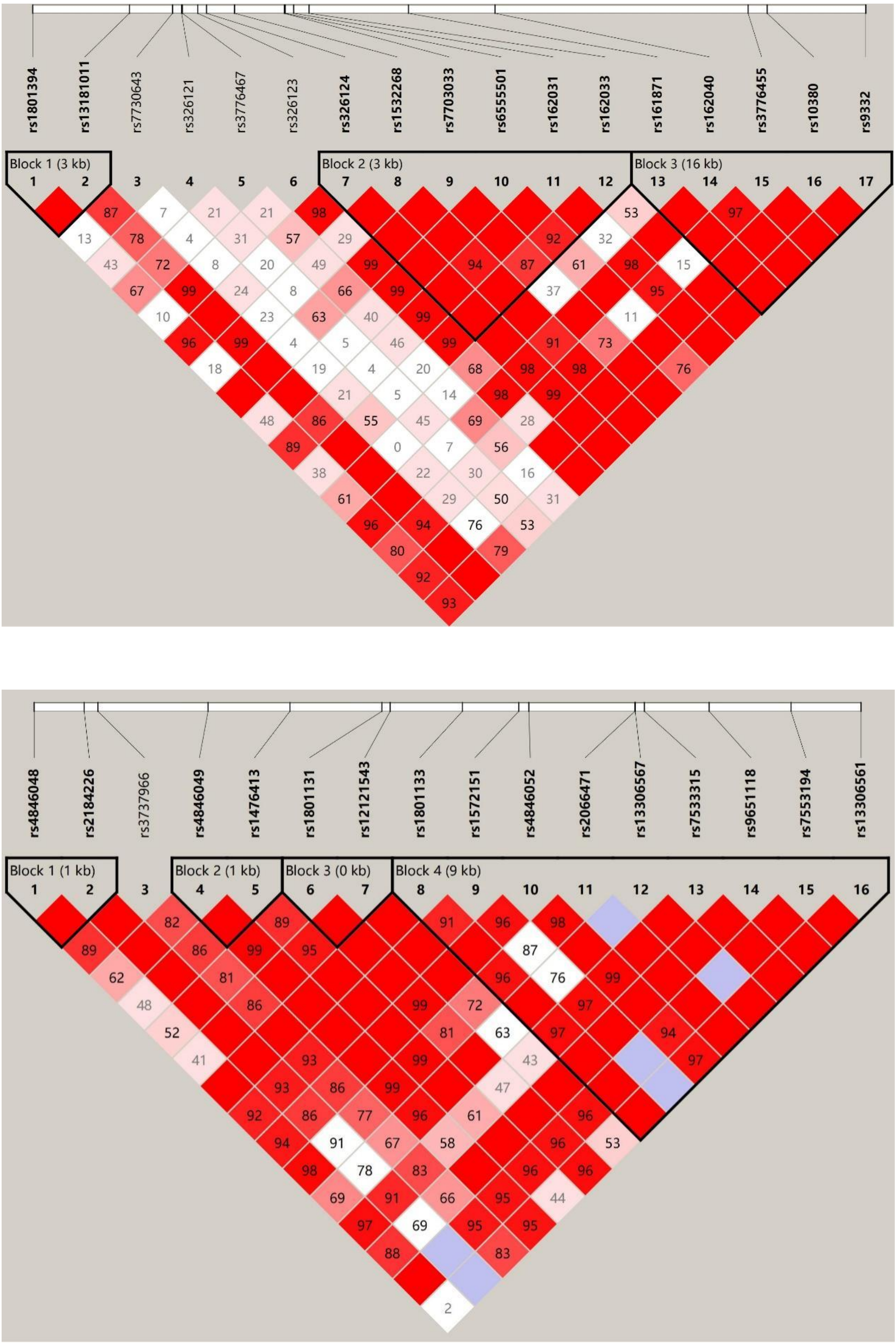

Figure S1. The linkage disequilibrium (LD) plot of (**A**) *MTRR* and (**B**) *MTHFR* genes. LD strength between the SNPs was indicated by the standard Haploview color scheme based on both D′ and LOD values (D′ < 1 and LOD < 2 in white; D′ = 1 and LOD < 2 in blue; D′ < 1 and LOD ≥ 2 in shades of pink/red; D′ = 1 and LOD ≥ 2 in bright red). Numbers in squares are D′ (×100), but those with D′

= 1 are not shown. The black triangle marks the single haplotype block within each gene. *MTRR*, methionine synthase reductase; *MTHFR*, methylenetetrahydrofolate reductase.
